# Beneficial Effects of Schisandrin B on the Cardiac Function in Mice Model of Myocardial Infarction

**DOI:** 10.1371/journal.pone.0079418

**Published:** 2013-11-08

**Authors:** Pengsheng Chen, Sisi Pang, Naiquan Yang, Haoyu Meng, Jia Liu, Ningtian Zhou, Min Zhang, Zhihui Xu, Wei Gao, Bo Chen, Zhengxian Tao, Liansheng Wang, Zhijian Yang

**Affiliations:** 1 Department of Cardiology, The First Affiliated Hospital of Nanjing Medical University, Nanjing, China; 2 Department of Geriatrics, The First Affiliated Hospital of Nanjing Medical University, Nanjing, China; 3 Department of Cardiology, Huai’an Second People’s Hospital Affiliated to Xuzhou Medical College, Huai’an, China; 4 Key Laboratory of Human Functional Genomics of Jiangsu Province, Clinical Diabetes Centre of Jiangsu Province, Nanjing Medical University, Nanjing, China; San Diego State University, United States of America

## Abstract

The fruit of Schisandra chinensis has been used in the traditional Chinese medicine for thousands of years. Accumulating evidence suggests that Schisandrin B (Sch B) has cardioprotection effect on myocardial ischemia *in* *vitro*. However, it is unclear whether Sch B has beneficial effects on continuous myocardial ischemia *in vivo*. The aim of the present study was to investigate whether Sch B could improve cardiac function and attenuate myocardial remodeling after myocardial infarction (MI) in mice. Mice model of MI was established by permanent ligation of the left anterior descending (LAD) coronary artery. Then the MI mice were randomly treated with Sch B or vehicle alone. After treatment for 3 weeks, Sch B could increase survival rate, improve heart function and decrease infarct size compared with vehicle. Moreover, Sch B could down-regulate some inflammatory cytokines, activate eNOS pathway, inhibit cell apoptosis, and enhance cell proliferation. Further *in vitro* study on H9c2 cells showed similar effects of Sch B on prevention of hypoxia-induced inflammation and cell apoptosis. Taken together, our results demonstrate that Sch B can reduce inflammation, inhibit apoptosis, and improve cardiac function after ischemic injury. It represents a potential novel therapeutic approach for treatment of ischemic heart disease.

## Introduction

Myocardial infarction (MI) is one of the most frequent causes of death. More than 20 percent of deaths are caused by coronary heart disease (CHD) [1]. Furthermore, even though patients could survive from acute MI, most of them inevitably suffered from heart failure (HF) [2]. And the most likely mechanism is left ventricular (LV) myocardial remodeling, which leads to functional decompensation and then HF [3], [4]. Since the prevalence, incidence and economic burden of HF are steadily increasing, it is necessary to explore efficient therapeutic measures to prevent myocardial remodeling induced by MI.

Fruits of Schisandra have been traditionally used in East Asia for the treatment of many uncomfortable symptoms, such as cough, dyspnea, dysentery, insomnia and amnesia for a long time [5], [6]. Schisandrin B (Sch B) is the most abundant dibenzocyclooctadiene derivative in Schisandra chinensis. Initially, Sch B was showed to have antioxidant effect on liver [7], [8]. In recent years, Sch B has been proved to have beneficial effect on ischemic diseases, such as cerebral ischemia and ischemia/reperfusion injury [9], [10]. Furthermore, it also has multiple cardioprotective effects, such as reducing cardiac toxicity caused by adriamycin and myocardial ischemia/reperfusion injury. And the structural determinants of this function maybe methylenedioxy group and cyclooctadiene ring [11]. The potential mechanism underlying the cardioprotective effects of SchB has been considered as the alleviation of oxidative stress [12], [13], [14], [15], [16]. However, it is unclear whether Sch B is still valid with regard to the cardioprotective action through other mechanisms.

Previous studies have confirmed that ischemia induced myocardial fibrosis, inflammation and apoptosis are essential factors in the process of LV myocardial remodeling after MI [17]. It has been demonstrated that some pro-inflammatory mediators play crucial roles in the pathogenesis of myocardial remodeling, such as transforming growth factor beta 1 (TGF-β1), tumor necrosis factor alpha (TNF-α), and interleukin 1 beta (IL-1β) [18], [19]. And suppression the expression of these factors can reduce the progression of myocardial remodeling. Some reports demonstrated that transcription factors, such as GATA4, Hand2, MEF2C and Tbx5, could reprogram cardiac fibroblasts into cardiomyocytes in *vitro*. These transcription factors can improve myocardial remodeling through cardiac repair [20]. Similarly, promoting endothelial nitric oxide synthase (eNOS) activation, which activates nitric oxide (NO), can induce angiogensis and improve myocardial ischemia after MI [21], [22]. Several studies *in* *vitro* have demonstrated that Sch B has anti-apoptotic, anti-inflammatory and anti-fibrotic activity [23], [24], [25]. It has also been proved that Sch B could restore eNOS in *vivo* [26]. Based on these reports, we hypothesized that administration of Sch B might have beneficial effect on the cardiac function after MI. Yet, whether treatment with Sch B has cardioprotective effects on continuous myocardial ischemia and the potential mechanisms remain to be determined. In the present study, we cultured H9c2 cells pre-treated with Sch B in different concentration before hypoxia stimulation to understand the potential mechanisms and the dose response. We examined the changes of inflammation and apoptosis related proteins and cell viability in Sch B pre-treated H9c2 cells after hypoxia stimulation. And we used mice model of continuous myocardial ischemia by permanent ligation of left anterior descending (LAD) coronary artery [27] to investigate whether treatment with Sch B could improve cardiac function and attenuate myocardial remodeling after MI in mice.

## Materials and Methods

### Animal

C57BL/6J male mice (aged 6–8 weeks, weighing 20−25 g) obtained from Laboratory Animal Center of Nanjing Medical University were employed in this study. The study complied with standards for the Care and Use of Laboratory Animals (Laboratory Animal Center of Nanjing Medical University). And the animal protocol was approved by the Ethics Review of Lab Animal Use Application of Nanjing Medical University (Permit Number: NJMU-ERLAUA-20120101).

### Mice Model of Myocardial Infarction

Briefly, mice were intraperitoneally anesthetized with pentobarbital sodium (50 mg/kg). After the anesthesia effected, mice were placed on a heating pad to maintain normothermia (about 35°C) and then fixed in the supine position by tying the legs and the upper jaw. Then they were intubated, and ventilated to perform a left thoracotomy to expose the heart. The electrocardiogram (ECG), heart rate and respiratory rate were continuously monitored. MI was induced by permanent ligation of the LAD coronary artery, using an 8-0 polypropylene suture passed about 2–3 mm from the inferior margin of left auricle. Ischemia was confirmed by myocardial blanching and ECG ST-segment elevation. Sham operations were carried out by the same method but without tying the suture on the LAD. In the process of operation, we dripped physiological saline on conjunctiva and cornea to prevent blindness caused by corneal drying in mice. The thorax was closed with standard produces. Then we gave intramuscular injection of penicillin and subcutaneous injection of analgesic. After restoring spontaneous breathing, mice were pulled out the endotracheal intubation, and then mice were placed on electric blanket waiting for their revival.

### Sch B Treatment Protocol *in vivo*

Sch B was purchased from the National Institute for the Control of Pharmaceutical and Biological Products (Beijing, China, purity >96%). The operated mice as described above survived for 6 hours were randomized to treatment with Sch B (80 mg/kg/day, intragastric administration, n = 20) or vehicle alone (n = 20) for 3 weeks. Sham-operated mice were also given the vehicle (n = 10). We observed the mice every 6 hours after the surgery and weighed the mice every two days. Mice were sacrificed by carbon dioxide (CO_2_) suffocated to death at 21^st^ days.

### Cell Culture and Induction of Hypoxia

H9c2 cells obtained from the Cell Bank of the Chinese Academy of Sciences were cultured in Dulbecco’s modified Eagle’s medium (DMEM) (GIBCO, Grand Island, NY, USA) supplemented with 10% fetal bovine serum at 37°C in a 95% air/5% CO_2_ atmosphere in 75 cm^2^ culture flasks. The medium was supplemented with streptomycin (100 µg/ml) and penicillin (100 IU/ml) and the medium was freshly replaced every 2 or 3 days. The cells were allowed to grow at 80% of confluence within 24 h prior to drug treatment. Previous studies indicated that pretreatment with Sch B for 16–24 h produced optimal myocardioprotection against ischemic injury *in vitro* [28]. After pre-treated with Sch B (5–20 µM) for 16 h, cells were washed and supplied with serum-free DMEM. DMSO (vehicle) was used as vehicle control. Establishment of hypoxic conditions was achieved by continuously flushing a chamber with a water saturated mixture of 5% CO_2_ and 95% N_2_ for 3 h.

### Echocardiography Measurement

Cardiac function was evaluated by using a high-frequency ultrasound system Vevo2100 (VisualSonics Inc, Toronto, ON, Canada) with a 30 MHz central frequency scan head. Mice were anesthetized with 1–2% isoflurane vapor in a 1∶1 air: oxygen mixture via a nose cone and positioned on a heating pad to maintain normothermia. The ECG, heart rate and respiratory rate were continuously monitored. Two-dimensional echocardiographic views of the midventricular short axis were obtained. From M-mode tracing, the left ventricular end-systolic diameter (LVESd), left ventricular end-diastolic diameter (LVEDd), left ventricular posterior wall end-systolic thickness (LVPWTs), left ventricular posterior wall end-diastolic thickness (LVPWTd), interventricular septum end-systolic thickness (IVSs) and interventricular septum end-diastolic thickness (IVSd) were measured. While left ventricular end-diastolic volume (LVEDV), left ventricular end-systolic volume (LVESV), left ventricular ejection fraction (LVEF) and left ventricular fractional shortening (LVFS) were obtained by the following formulas [29]:
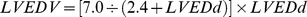


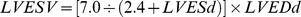


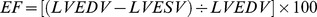


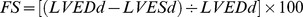



### Measurement of Myocardial Infarction Size

At 21^st^ days after operation, mice were intraperitoneally anesthetized with pentobarbital sodium (50 mg/kg). 1 ml Evans blue (0.1 g/ml; BioSharp, China) was slowly injected into abdominal aorta and then the heart was removed immediately and weighed. After stored for 20 minutes at −20°C, the heart was cut into 4 or 5 transverse slices (1–2 mm thickness) across the long axis. Then the slices were stained with 1% triphenyltetrazolium chloride (TTC, Amresco, USA) in citrate buffer solution (ph = 7.4) for 30 min at 37°C to delineate the area at risk. And then all slices fixed 4% paraformaldehyde for overnight. Each slice was photographed and analyzed. The infarct area was pale white while the non-infarct but at risk area was red. The infarcted to risk ratios were calculated by computerized planimetry (Image J, version 1.44, NIH, Bethesda, MD).

### Histopathology

To evaluate the morphological changes and the extent of cardiac fibrosis, the hearts were harvested, washed in PBS and fixed in 4% paraformaldehyde for overnight and embedded in paraffin at 21^st^ days after MI. Each heart was cut into sections of 4 µm-thick and stained with hematein and eosin (H&E) and Masson trichrome. And each section was imaged by a microscopy (Nikon, Japan).

### Detection of BrdU by Immunohistochemistry

To evaluate whether there was proliferation in cardiac muscle, mice were intraperitoneally injected bromodeoxyuridine (BrdU, 100 mg/kg, Sigma, St Louis, USA) once a day for 2 consecutive days before sacrificed. Then the myocardial tissues were fixed with 4% paraformaldehyde and paraffin embedded. After antigen were retrieved in citrate buffer (pH 6.0), the sections were incubated with anti-BrdU(1∶200,Sigma, St Louis, USA). Nuclei were stained with DAPI (1 µg/ml; Sigma, St Louis, USA). Then they were applied followed by Alexa Fluor®635 donkey anti-mouse (1∶200; Invitrogen, Eugene, USA). Image-Pro Plus software (Media Cybernetics, Rockville, USA) was used to determine the area of BrdU and DAPI-positive staining.

### Measurement of Apoptotic Cells by TUNEL Staining

At 21^st^ days after operation, the number and distribution of apoptotic cells were detected by using an apoptosis *in situ* Cell Death Detection Kit (Biouniquer, Nanjing China) according to the instructions provided by the manufacturer. All slices were stained with DAPI (1 µg/ml; Sigma, St Louis, USA) for the assessment of nuclear morphology. The FITC-labeled TUNEL-positive cells were imaged by a fluorescent microscopy at 400×magnification (Nikon, Japan) and five horizons were randomly selected in each section. The characterization of apoptosis induced by hypoxia and serum deprivation in H9c2 cells was performed using the *in situ* Cell Death Detection Kit. The FITC-labeled TUNEL-positive cells were counted using Image-Pro Plus software (Media Cybernetics, Rockville, USA). 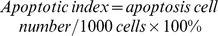.

### Western Blotting Analysis

Total protein *in vivo* was obtained from left ventricular myocardial tissues. H9c2 cells were lysed in Lysis Buffer containing protease inhibitor and phosphatase inhibitor. After centrifugation, they were followed by sonication and heat denaturation. A total of 20 ug protein lysates were electrophoresed and separated on 6%–12% SDS-PAGE and transferred onto nitrocellulose membranes (Bio-Rad, Hercules, USA). The membranes were blocked with 5% skim milk at room temperature for an hour, and then incubated over night at 4°C with primary antibodies including rabbit anti-eNOS (1∶1000; Sigma, St Louis, USA), rabbit anti-phospho-eNOS (1∶200; Santa Cruz Biotechnology, Santa Cruz, USA), rabbit anti-NF-κB (1∶800; Cell Signaling Technology, USA), rabbit anti-Bcl-2 (1∶800; Bioworld, USA), rabbit anti-Bax (1∶800; Bioworld, USA), rabbit anti-ASK1 (1∶1000; Cell Signaling Technology, USA) and rabbit anti-GAPDH (1∶1000; Cell Signaling Technology, USA). The membranes were then incubated with HRP-conjugated secondary antibodies (1∶500; Santa Cruz Biotechnology, Santa Cruz, USA) at room temperature for 1 hour. The antigen–antibody complexes were detected by using a SuperSignal ECL kit (Thermo, USA) in a Western blotting detection system (Bio-Rad, CA, USA). Results were expressed as density values normalized to GAPDH.

### ELISA Measurement of TGF-β1, TNF-α and IL-1β

ELISAs for TGF-β1, TNF-α and IL-1β (Bio-Swamp, Shanghai, China) were conducted on myocardial tissue lysates from left ventricle. Briefly, 20 mg myocardial tissue samples were homogenized in 200 ul of 1× PBS (pH = 7.4), then stored overnight at −20°C. After two freeze-thaw cycles to break the cell membranes, the homogenates were centrifuged at 5000 *g* for 10 minutes. Then samples were assayed immediately following the procedure recommended by the manufacturer.

### Cell Viability Assay

A 3-(4,5-dimethylthiazol-2-yl)-2,5-diphenyltetrazolium bromide (MTT) assay was used to determine cell viability. The cells were plated on 96-well plates and MTT was added at a final concentration of 0.5 mg/ml, followed by incubation for 4 h at 37°C. Absorbance was measured using a microplate reader (Bio-Rad, CA, USA) at a test wavelength of 570 nm. The cells incubated with control medium were considered to be 100% viable. The percentage of cell survival was calculated by the ratio of mean absorbance in test wells and mean absorbance in control wells.

### Statistical Analysis

Data were expressed as mean ± standard error of mean (SEM) and p-value <0.05 considered statistically significant. Differences were determined using two-tailed Student’s t test or one-way ANOVA, and Newman–Keuls test was performed to determine post hoc differences. The overall survival of mice after MI was evaluated using Kaplan-Meier curves survival analysis and compared by log-rank test. All statistical analyses were conducted using SPSS 13.0 or GraphPad Prism 5.

## Results

### Sch B Treatment Improves Survival Rate and Cardiac Function in Mice after MI

**Figure 1:**
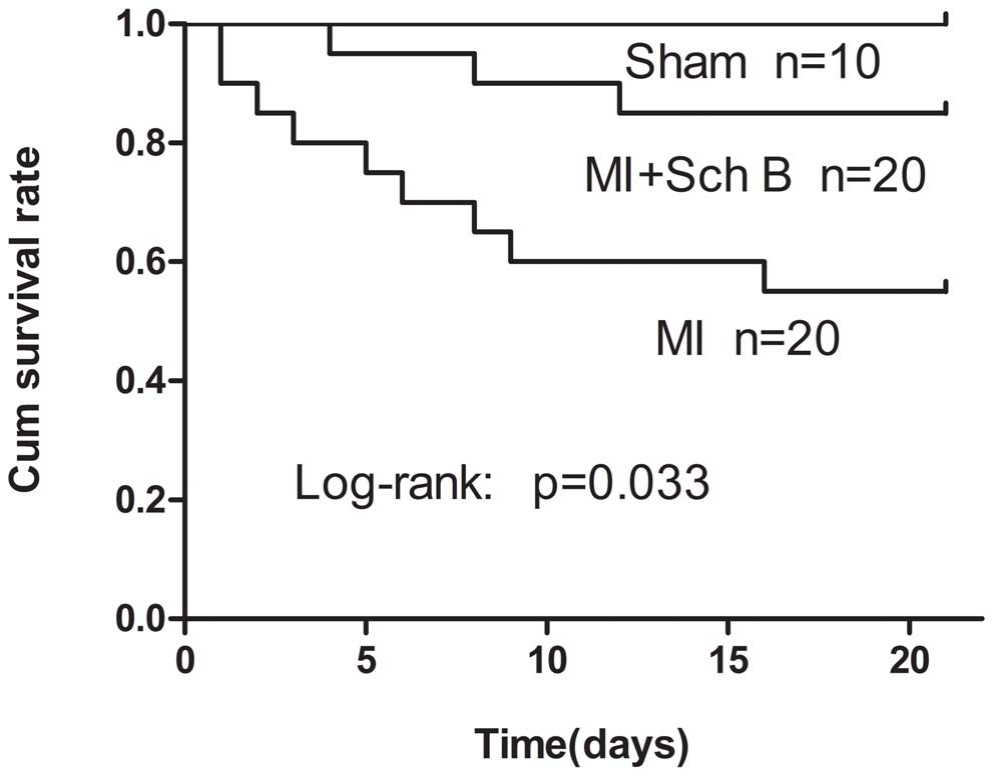
Survival rate at 3 weeks after myocardial infarction (MI). Kaplan-Meier analysis showed significantly lower mortality in Schisandrin B (Sch B) treated mice after MI than in untreated MI mice (log-rank : *P* = 0.033).

**Figure 2:**
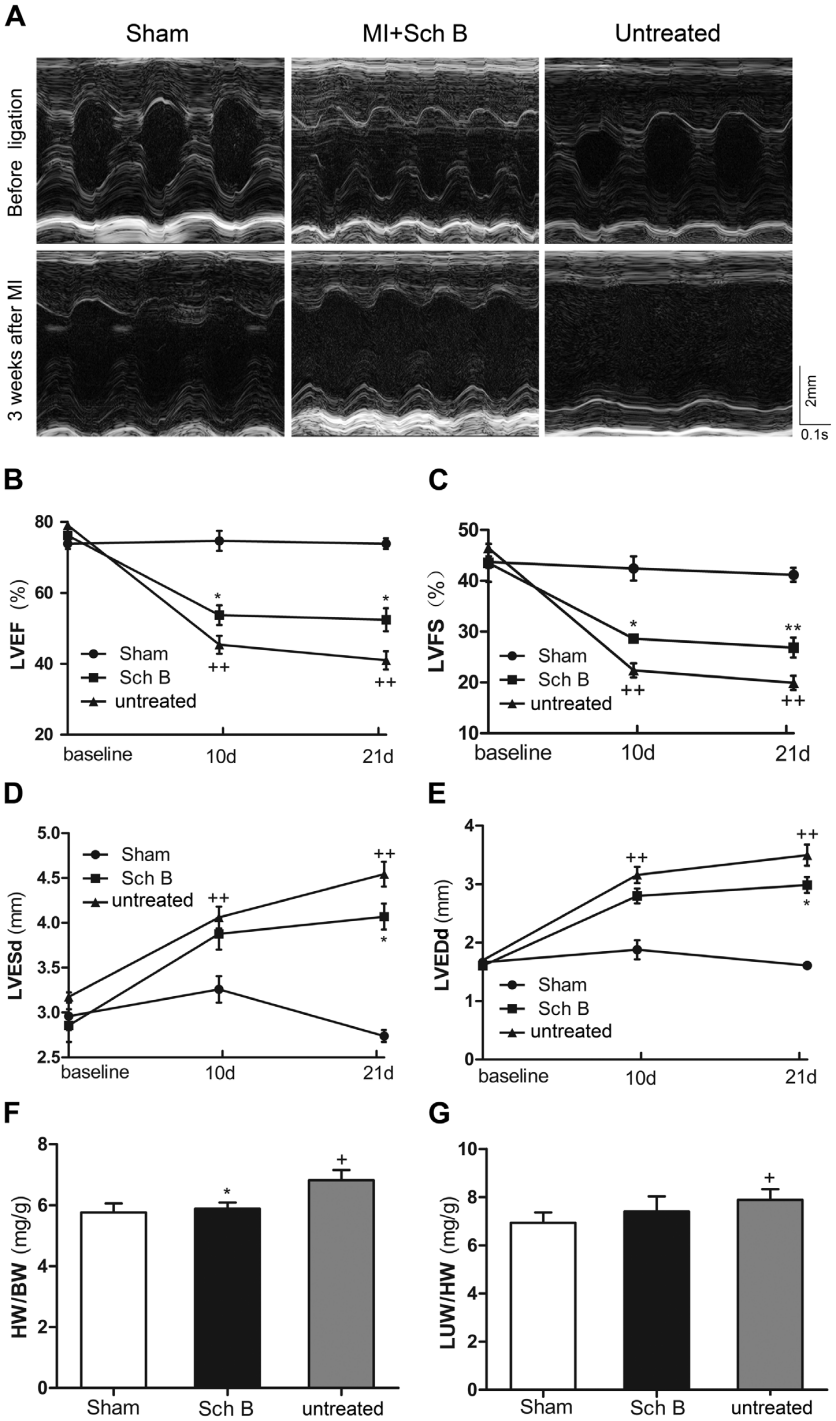
Sch B in the effects of cardiac remodeling in MI mice. (A) Representative M-mode echocardiographic images of mice. (B) Analysis of left ventricular ejection fraction (LVEF) at 10 d and 21 d after MI. (C) Analysis of left ventricular fractional shortening (LVFS) at 10 d and 21 d after MI. (D) Analysis of left ventricular end-systolic diameter (LVESd) at 10 d and 21 d after MI. (E) Analysis of left ventricular end-diastolic diameter (LVEDd ) at 10 d and 21 d after MI. (F) Analysis of heart weight/body weight ratio (HW/BW) at 10 d and 21 d after MI. (G) Analysis of the lung weight/body weight ratio (LUW/BW) at 10 d and 21 d after MI. n = 8 in the sham-operated group, n = 10 in the Sch B treated and untreated groups. *P<0.05 versus untreated group, **P<0.01 versus untreated group, +*P*<0.05 versus sham-operated group, ++*P*<0.01 versus sham-operated group.

After MI, improved survival was observed in mice treated by Sch B when compared with those untreated mice. Postmortem examination indicated that the main cause of death was HF. The survival rate of Sch B treated group was 85% and the untreated group was 55% (log-rank test, *P* = 0.033; **Fig. 1**). Echocardiography of Sch B-treated group showed a significant decrease in LVESd and LVEDd and an increase in LVEF and LVFS, compared with the untreated group [**Fig. 2** **(A–E)**]. After 3 weeks of MI, the heart weight/body weight (HW/BW) ratio was significantly lower in Sch B-treated mice than in untreated mice [**Fig. 2** **(F)**]. Although Sch B-treated mice had a trend of lower lung weight/body weight ratio (LUW/BW) than untreated mice, the difference did not reach statistical significance [**Fig. 2** **(G)**].

### Sch B Treatment Reduces Infarct Size

**Figure 3:**
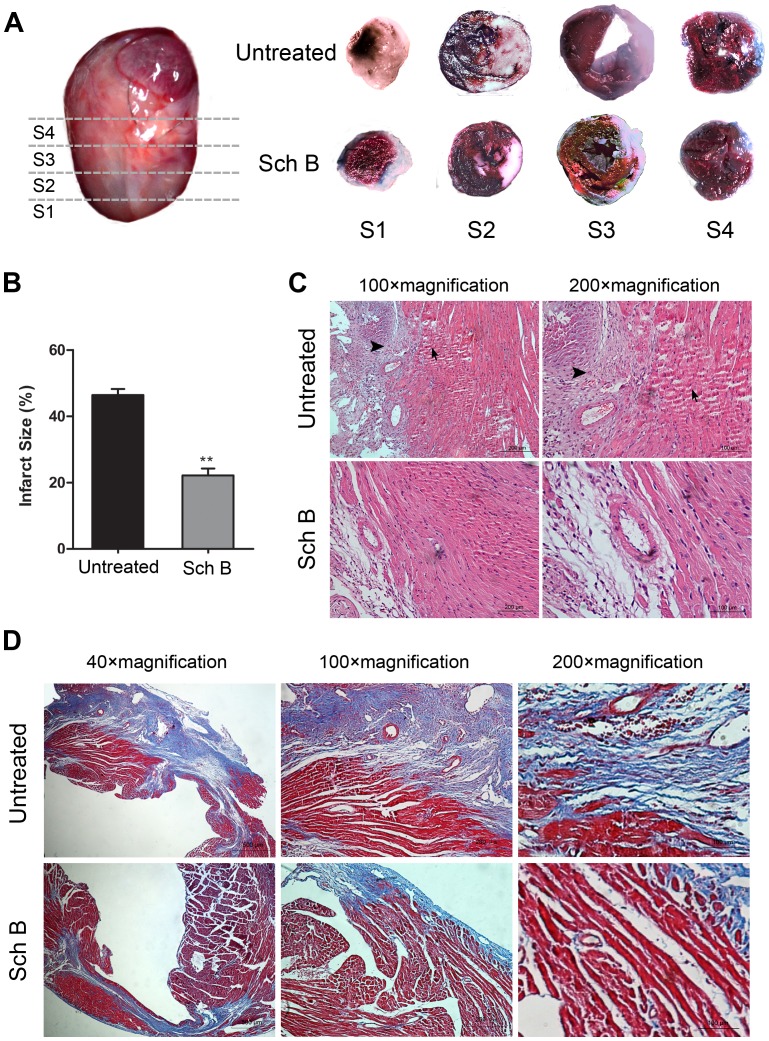
Pathological changes of the morphology in Sch B treated group and the untreated group in MI mice. (A) Hearts were cut transversely into 4 slices at 3 weeks after MI. Each slice was stained with Evans blue and triphenyltetrazolium chloride (TTC). Viable myocardium stains red, while the infarct area stains pale. (B) Infarct size analysis of Sch B treated group and the untreated group. Infarct size expressed as percentage infarction of the area at risk in hearts. Area at risk as percentage of the area of whole left ventricle didn’t differ between the two groups. (C) Representative illustration of hematoxylin and eosin staining of infarcted mouse hearts. These photos demonstrated the intense inflammatory response (**arrowheads**) and myocardial cells arranged irregularly (**arrows**) after MI. (D) Representative images of Masson’s Trichrome-stained infarcted hearts in mice. Blue represents region with replacement fibrosis. n = 6 per group, *P<0.05 versus untreated group, **P<0.01 versus untreated group.

Evans blue and TTC staining were used to detect the size of infarct area and non-infarct but at risk area. The risk areas of the total LV were similar between Sch B treated group and the untreated group. While treatment with Sch B was associated with a significantly reduced infarct size compared to the untreated group [**Fig. 3** **(A, B)**].

### Sch B Treatment Improves Pathological Changes in Myocardial Tissue

After 3 weeks, as shown by H&E staining, in the untreated group, the surviving myocardial cells were found in the border zones and arranged irregularly. And there was much infiltration of inflammatory cells. Contrastly, in the treated group, most of myocardial cells were normal and arranged in an orderly manner, and the area of necrosis was smaller compared to the untreated group [**Fig. 3** **(C)**]. The analysis of Masson trichrome staining demonstrated that treatment with Sch B could significantly decrease fibrosis and collagen deposition after MI compared to the untreated group [**Fig. 3** **(D)**].

### Sch B Treatment Suppresses Expression of Inflammatory Cytokines

**Figure 4:**
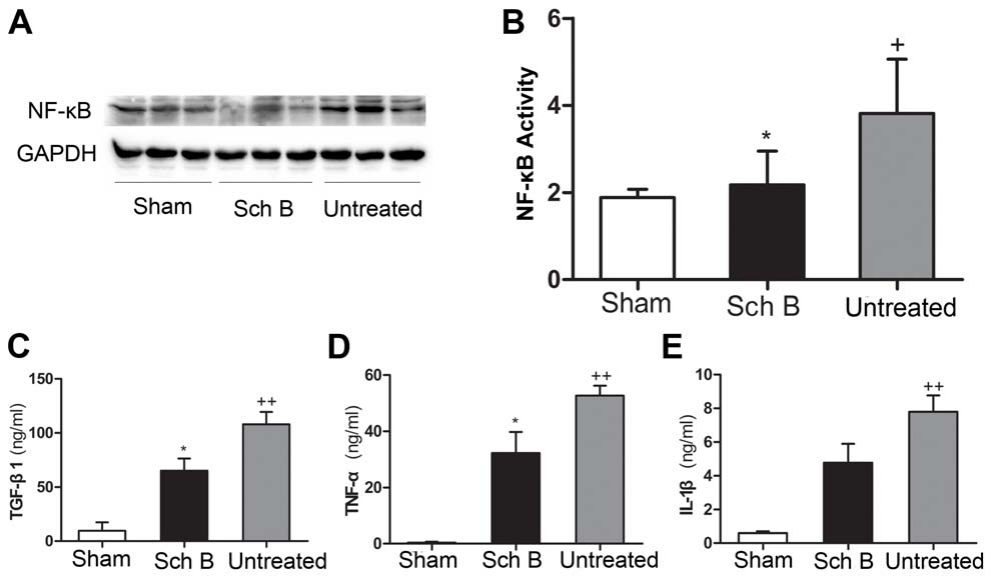
Sch B in the effects of expression of inflammation cytokines after MI. (A) Western blot analysis of NF-κB. GAPDH was used as an internal control. (B) Densitometry for NF-κB expression normalized to GAPDH. (C) Quantitative analysis of TGF-β1 expression of LV myocardium by ELISA. (D) Quantitative analysis of TNF-α expression of LV myocardium by ELISA. (E) Quantitative analysis of IL-1β expression of LV myocardium by ELISA. n = 8 per group, *P<0.05 versus untreated group, +*P*<0.05 versus sham-operated group, ++*P*<0.01 versus sham-operated group.

Expression levels of TGF-β1, TNF-α and IL-1β were determined in LV myocardial tissue lysates by ELISA. And the expression of NF-κB was determined by western blotting analysis. Compared with the sham-operated mice, the expression levels of TGF-β1, TNF-α, IL-1β and NF-κB were much higher in the untreated group, indicating that MI resulted in up-regulating pro-inflammation cytokines. The expression levels of TGF-β1 and TNF-α were significantly lower in the Sch B treated group than in the untreated group [**Fig. 4** **(A, B, C, D)**]. Although the Sch B treated group showed a trend of lower expression level of IL-1β than the untreated group, the difference did not reach the significant level **[** **Fig. 4** **(E)]**.

### Sch B Treatment Decreases Myocardium Apoptosis

**Figure 5:**
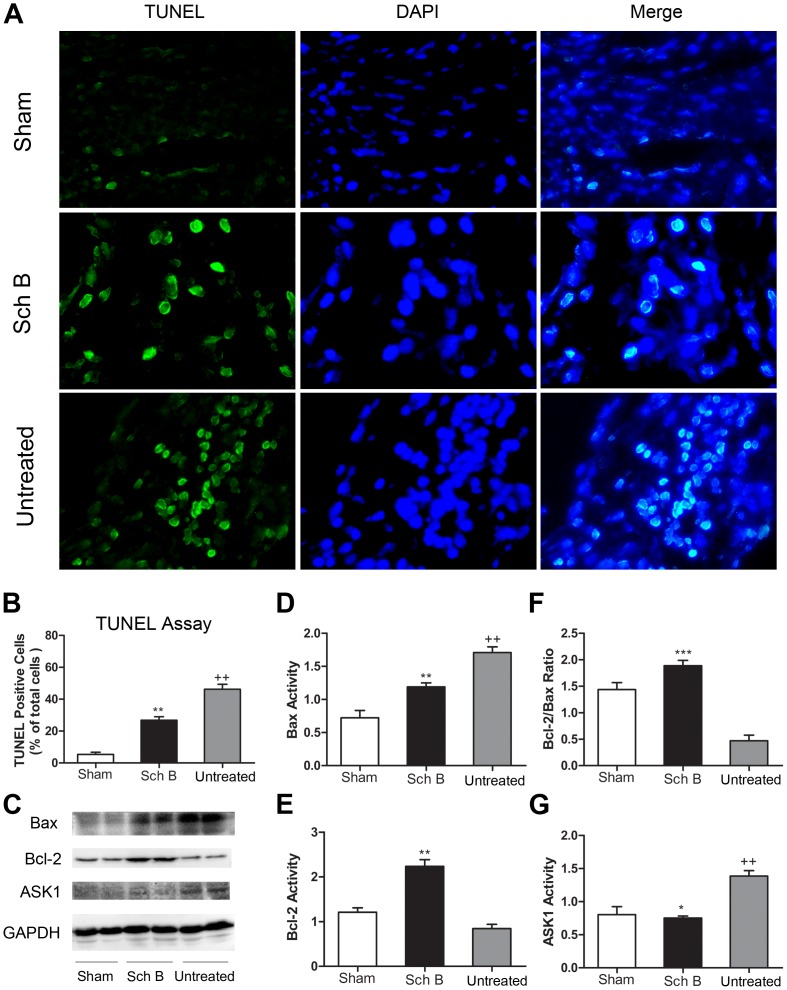
Sch B in the effects of myocardial cell apoptosis. (A) TUNEL analysis of the sham-operated mice, Sch B treated mice and the untreated mice at 3 weeks after MI. The green, TUNEL-positive cells are seen merged with blue DAPI-positive cells at 400×magnification. (B) The cardiomyocyte apoptosis index (number of TUNEL-positive cells per 1000 cells) was significantly lower in the Sch B treated group than in the untreated group. (C) Western blotting analysis of Bax, Bcl-2 and ASK1. GAPDH was used as an internal control. (D) Densitometry for Bax expression normalized to GAPDH. (E) Densitometry for Bcl-2 expression normalized to GAPDH. (F) Analysis of the ratio between the expression of Bcl-2 and Bax. (G) Densitometry for ASK1 expression normalized to GAPDH. n = 6 per group, *P<0.05 versus untreated group, **P<0.01 versus untreated group, ***P<0.001 versus untreated group, +*P*<0.05 versus sham-operated group, ++*P*<0.01 versus sham-operated group.

At 21^st^ days after operation, apoptosis levels in the border regions of myocardium were determined among the three groups. The apoptotic index (number of TUNEL-positive cells per 1000 cells) in ischemic myocardium tissue of untreated group was significantly higher than in the sham-operated group. When treated with Sch B, the number of TUNEL-positive cells in the border regions of the infarcted myocardium was significantly smaller compared with the untreated group [**Fig. 5** **(A, B)**]. To examine the effect of Sch B on apoptosis related protein expression levels, we determined the expression of Bax, Bcl-2 and apoptosis signal-regulating kinase 1 (ASK1) by western bolting analysis. We found that the expression of Bax was significantly decreased while Bcl-2 expression was significantly increased in treated group, leading to an increased Bcl-2/Bax ratio compared to untreated group [**Fig. 5** **(C, D, E, F)**]. And treatment with Sch B led to a significant down-regulation of ASK1 expression levels [**Fig. 5** **(C, G)**].

### Sch B Treatment Activates eNOS Signaling and Up-regulated Gata4

**Figure 6:**
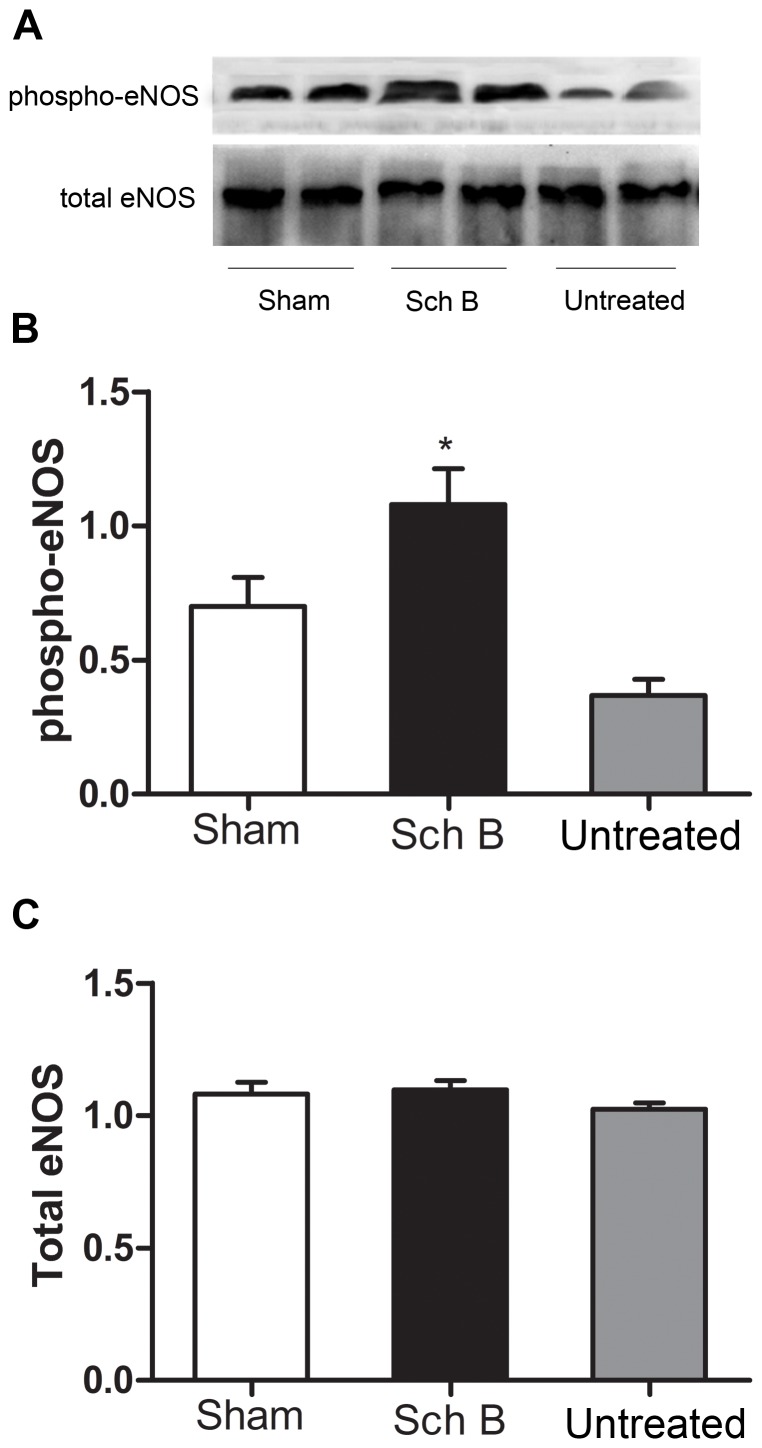
Analysis of eNOS expression in LV myocardium of mice. (A) Western blot analysis of total eNOS and phospho-eNOS Ser1177 expression. (B) Densitometry for phospho-eNOS expression. (C) Densitometry for total eNOS expression. n = 6 per group, *P<0.05 versus untreated group, +*P*<0.05 versus sham-operated group.

**Figure 7:**
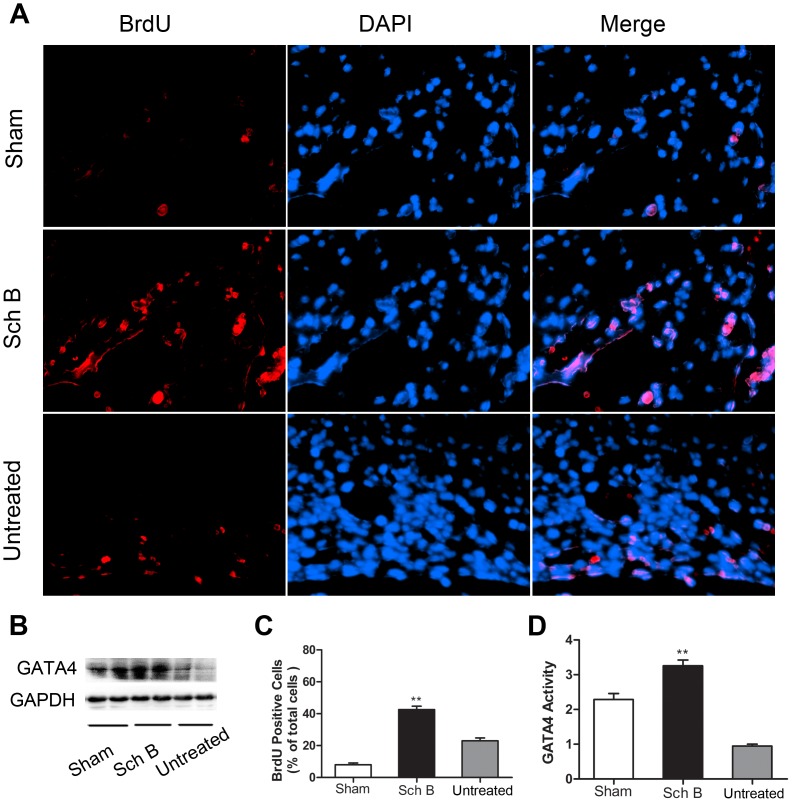
Sch B in the effects of enhancing proliferation and cardiac repair. (A) BrdU (red) and DAPI (blue) staining of ischemic myocardial tissue at 21 days after MI at 400×magnification. (B) Western blot analysis of GATA4. GAPDH was used as an internal control. (C) Analysis of the ratio between BrdU-stained cells and DAPI-stained cells in each group. (D) Densitometry for GATA4 expression normalized to GAPDH. n = 6 per group, **P<0.01 versus untreated group.

We determined whether Sch B treatment affected eNOS protein expression or phosphorylation in ischemic myocardium. Total eNOS expression in ischemic myocardium was similar among three groups. However, the expression of S1177- phosphorylated eNOS protein was significantly higher in the Sch B treated group [**Fig. 6**]. Similarly, the expression of GATA4 was significantly higher in Sch B treated group compared with the untreated group [**Fig. 7** **(B, D)**].

### Sch B Treatment Enhances Cell Proliferation after Myocardial Infarction

Proliferating cells were determined by BrdU and DAPI labeling. The rate of proliferating cells is expressed as a percentage of BrdU/DAPI double-positive cells versus DAPI positive cells [**Fig. 7** **(A)**]. The number of BrdU-positive/DAPI-positive cells was significantly higher in the treated group compared with the untreated group [**Fig. 7(C)**]. And the BrdU-positive cells appeared to be replicating at the border of the infarct area and migrating inward.

### Sch B Treatment Suppresses Apoptosis and Inflammation in H9c2 Cells after Hypoxia Stimulation

**Figure 8:**
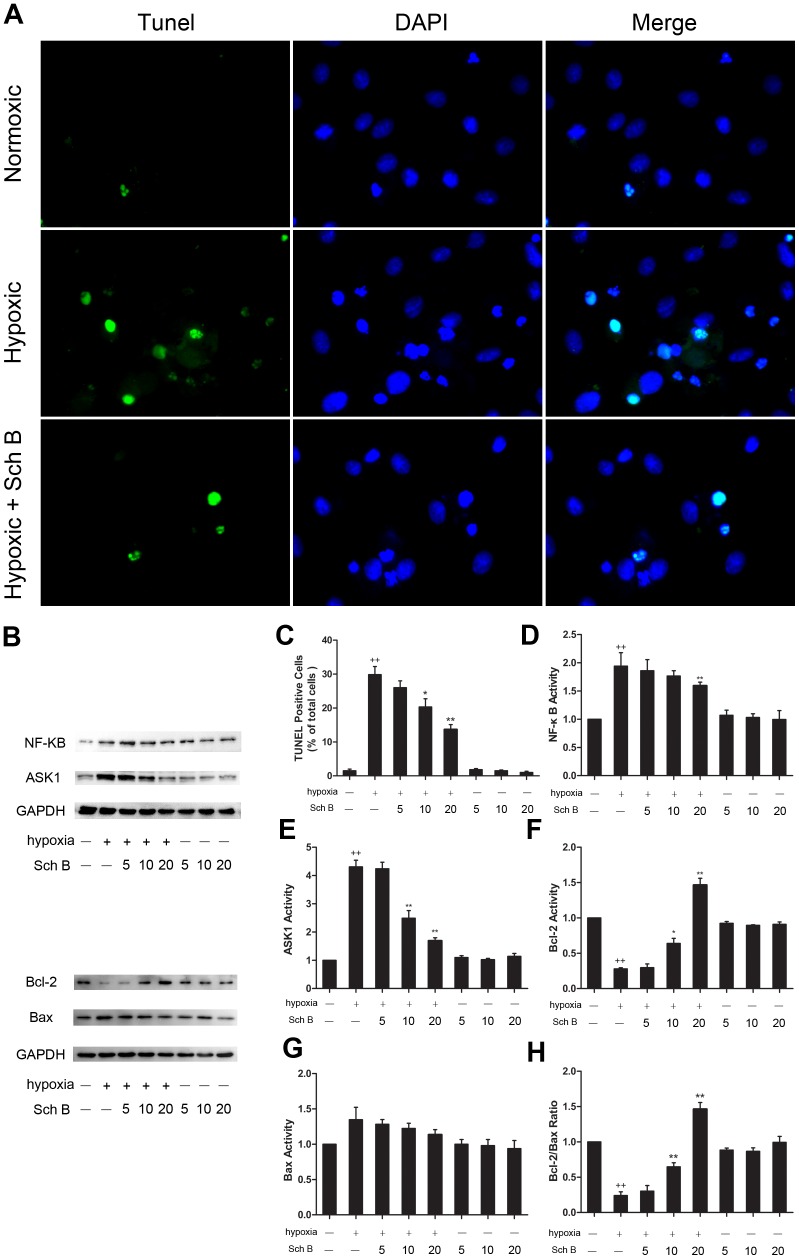
Effects of Sch B on H9c2 cells against hypoxia induced apoptosis and inflammation. (A) Representative images of TUNEL staining in H2c9 cells after hypoxia or normoxia. TUNEL-positive cells were green and DAPI-positive cells are blue. (B) Western blotting analysis of NF-κB, ASK1, Bcl-2 and Bax. GAPDH was used as an internal control. (C) Analysis of apoptotic index after TUNEL staining for each group. Cells were treated as follows: three different concentrations of Sch B (5, 10 and 20 µM) with or without hypoxia stimulation. (D) Densitometry for NF-κB expression normalized to GAPDH. (E) Densitometry for ASK1 expression normalized to GAPDH. (F) Densitometry for Bcl-2 expression normalized to GAPDH. (G) Densitometry for Bax expression normalized to GAPDH. (H) Analysis of the ratio between the expression of Bcl-2 and Bax. n = 3 per group, *P<0.05 versus hypoxia (+) and Sch (−) group, **P<0.01 versus hypoxia (+) and Sch (−) group, +*P*<0.05 versus hypoxia (−) and Sch (−) group, ++*P*<0.01 versus hypoxia (−) and Sch (−) group.

*In vitro* study, after pre-treatment with Sch B for 16 h and then hypoxia for 12 h, the TUNEL staining showed that pre-treated with Sch B could significantly reduce the positive rate of apoptotic cells in a concentration-dependent manner [**Fig. 8** **(A, C)**]. The expression of apoptosis and inflammation related proteins were also detected. Similarity to the study *in vivo*, the expression of Bcl-2 was increased while the expression of Bax was decreased after hypoxia stimulation in H2c9 cells pre-treated with Sch B in a concentration-dependent manner [**Fig. 8** **(B, F, G, H)**]. In addition, cells pre-treated with Sch B also showed a significant decrease in the expression of NF-κB and ASK1 in a concentration-dependent manner **[** **Fig. 8** **(B, D, E)]**.

### Sch B Increased the Viability of H9c2 Cells and the Expression of GATA4 *in vitro*

**Figure 9:**
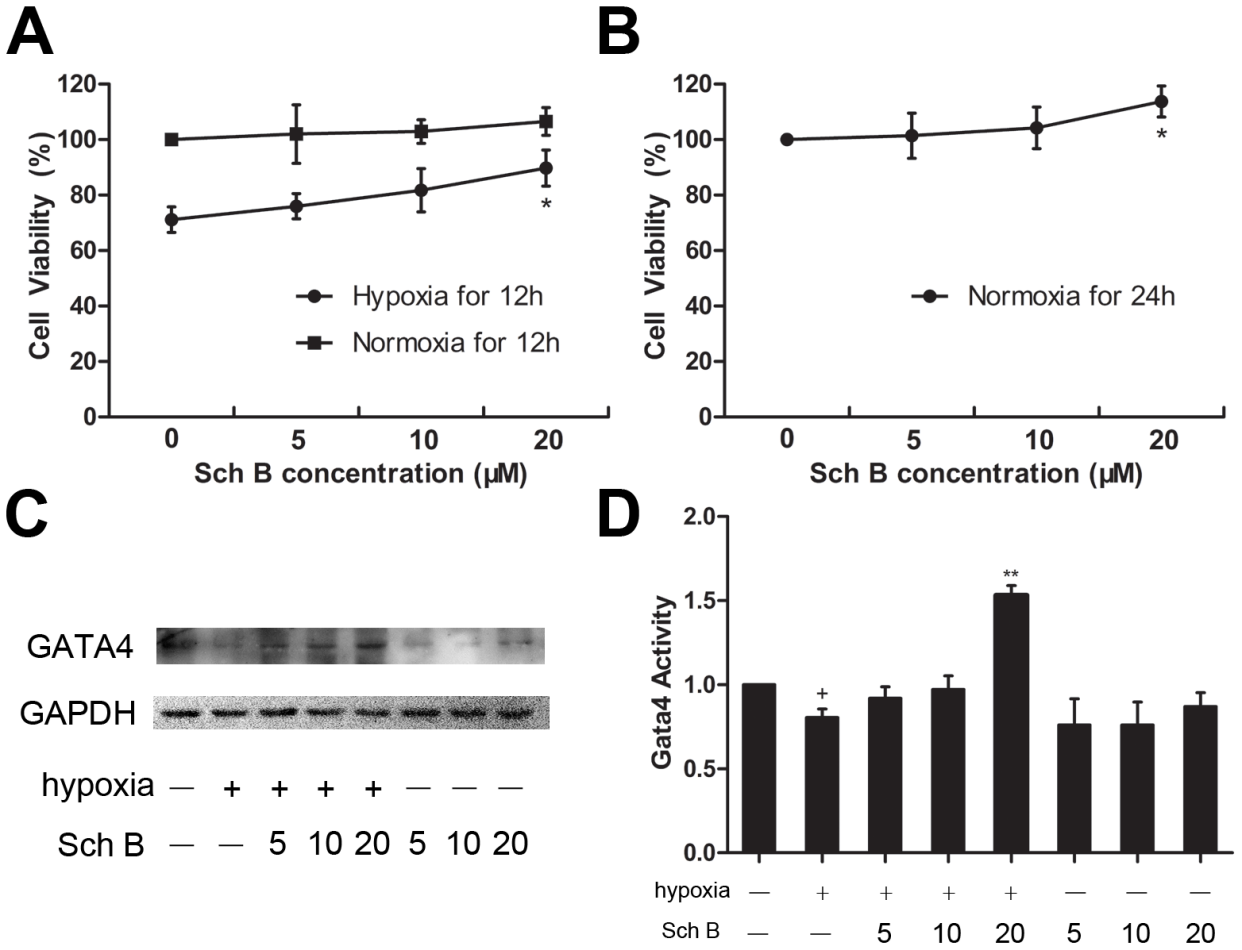
Sch B in the effects of increasing cell viability in H9c2 cells. (A) Cell viability determined by MTT assay after hypoxic injury or normoxic for 12 hours pre-treatment with Sch B induced concentrations-dependent increases in survival of H9c2 cells. (B) Cell viability determined by MTT assay for 24 hours in normoxia condition pre-treatment with Sch B induced concentrations-dependent increases in survival of H9c2 cells. n = 6 per group, *P<0.05 versus 0 µM Sch B group. (C) Western blot analysis of GATA4. (D) Densitometry for GATA4 expression normalized to GAPDH. n = 3 per group, *P<0.05 versus hypoxia (+) and Sch (−) group, **P<0.01 versus hypoxia (+) and Sch (−) group, +*P*<0.05 versus hypoxia (−) and Sch (−) group, ++*P*<0.01 versus hypoxia (−) and Sch (−) group.

To study the effects of various concentrations of Sch B on H9c2 cells, we determined the number of viable cells with MTT. Compared with the normoxic cells, the viability of H9c2 cells in the hypoxic group was significantly decreased. When H9c2 cells were pre-treated with 5, 10, and 20 µM Sch B, they showed concentration-dependent increase in the number of viable cells. Among these, pre-treated with 20 µM Sch B resulted in significant increases in cell viability [**Fig. 9** **(A, B)**]. The effects of Sch B on up-regulating the expression of GATA4 were also verified. The expression of GATA4 was significantly decreased after hypoxic stimulation. However, when pre-treated with Sch B, the expression of GATA4 in H9c2 cells was increased compared to the untreated group. [**Fig. 9** **(C, D)**].

## Discussion

In our study, we observed that treatment with Sch B in mice after MI could increase survival rate, improve heart function and decrease infarct size compared to the control group. And administration of Sch B after MI could attenuate myocardial fibrosis, inflammatory and apoptosis. These effects sequentially retard the progression of myocardial remodeling after MI. Furthermore, in our *in vitro* study, pre-treatment with Sch B in H9c2 cells could alleviate hypoxia induced inflammation and apoptosis. The potential mechanisms might be associated with down-regulated expression of TGF-β1, TNF-α, IL-1β, ASK1, NF-κB Bax, up-regulated expression of Bcl-2 and Gata4, activation of eNOS pathway to ameliorate myocardial ischemia, and enhanced cardiac repair. These findings indicate that Sch B could be an effective, preventive and therapeutic drug against progression of heart remodeling after MI.

After myocardial ischemia induced by coronary artery ligation, global expression of inflammatory markers increased in mice. The pro-inflammatory cytokines, including TGF-β1, TNF-α, and IL-1β, play crucial roles in myocardial fibrosis and the pathological progression of LV remodeling by inducing inflammatory action via the NF-κB pathway [30]. Moreover, Oxidative stress in acute MI trigger this process in chain with the pro-inflammatory cytokines. And oxidative stress stimulates expression of ASK1 and secondary activates NF-κB, leading to produce TNF-α [31], [32]. The overexpression of these inflammatory cytokines activates myocardial hypertrophic response, then myocardial remodeling and HF [33]. In our study, we found that the expression of inflammatory mediators were increased in MI mice and then caused myocardial remodeling, which was in line with the previous studies. It has also been demonstrated that Sch B had anti-inflammatory and anti-fibrotic activity in several studies in *vitro* [23], [24], [25]. Consistent with these findings, our data showed that administration of Sch B could decrease the expression of TGF-β1, TNF-α, NF-κB and ASK1 in a concentration-dependent manner. Our results indicate that Sch B therapy may reduce cardiac remodeling via anti-inflammatory, anti-fibrotic as well as anti-oxidative stress.

The present study demonstrates that treatment with Sch B can enhance the anti-apoptotic effect [24]. Apoptosis plays an important role in cardiovascular diseases. Recent studies have demonstrated that ischemic-induced apoptosis and necrosis contribute to autophagic cardiomyocyte death and cardiomyocyte loss in myocardial ischemic injury [34], [35]. In acute MI, the inflammatory cytokines in injured myocardium, e.g. TNF-α and IL-1β also stimulate apoptosis contributing to the process of myocardial remodeling [36]. Consistently, we found that apoptotic cells were prominent in MI mice. And the apoptotic index in border regions of infarcted myocardium after MI correlates well with previous studies [37], [38], [39]. Our data also indicate that Sch B treatment may have the capability of reducing apoptosis in a mice model of MI as well as a hypoxia model in H2c9 cells. The mechanism underlying this phenomenon might include up-regulated expression of Bcl-2 and suppressed expression of Bax, leading to an increased Bcl-2/Bax ratio which has been proved to play an important role in the regulation of cell apoptosis [40], [41]. Our finding correlates well with previous studies, and provides further evidence that Sch B exerts anti-apoptotic functions in infarct expansion during LV remodeling after myocardial ischemic injury.

GATA4 is the early cardiac transcription factor which is able to reprogram cardiac fibroblasts. This factor can further enhance the degree of cardiac repair and improve cardiac function after MI [42], [43]. It has been shown that up-regulating of GATA4 by reprogramming mesenchymal stem cells can induce extensive survival through attenuation of infarct size in mice model with acute MI [44]. In line with these studies, our data showed that Sch B treatment stimulated the expression of GATA4 and reduced the mortality as well as infarct size after MI. Moreover, Qian et al [45] demonstrated that GATA4 plays a role of cardiac repair by reprogramming resident nonmyocytes in the heart into newly born cardiomyocyte-like cells. Interestingly, our data showed the proliferation marker BrdU dyed cells appeared to be replicating at the border of the infarct area and migrating inward. Based on cardiac function improving in our MI mice, we hypothesize that these proliferating cells could regenerate muscle fibers or improve angiogenesis. Cells viability detected by MTT assay revealed that cell proliferation might be produced by the anti-apoptotic effect of Sch B. These results indicate that Sch B treatment has an ability of cardiac repair and enhancing cells proliferation after MI. Future studies are needed to identify the specific composition of these proliferated cells.

Several studies have shown that eNOS Ser1177 phosphorylation is associated with cardioprotective effects after myocardial ischemia [46]. In our study, we observed that administration of Sch B stimulated eNOS phosphorylation and significantly increased the level of p-eNOS in mice after MI. It has been proved that activation of eNOS pathway can induce eNOS to be phosphorylated and then synthesizes NO [47]. Both eNOS and NO can protect cardiac function via regulation of vascular remodeling and angiogenesis [48]. These data indicate that the activation of eNOS pathway when treated with Sch B may contribute to the protective effects on the infarcted myocardium.

To the best of our knowledge, our study is the first to report that Sch B treatment can reduce the mortality of mice with MI. Moreover, Sch B therapy may contribute to long-term improvement of LV function after MI by inhibiting myocardial fibrosis, inflammatory, apoptosis and enhancing cardiac repair. However, our study still has several limitations. First, although treatment with Sch B has a significant improvement in LV function, only parts of the potential underlying mechanisms were examined in the present studies. The therapeutic effects of Sch B may involve multiple pathways and further studies are needed to demonstrate additional pathways. Second, whether a combination of Sch B and other medications could achieve better therapeutic effects needs to be further investigated.

In conclusion, we have shown that administration with Sch B has notable benefits on cardiac function to reduce mortality and attenuates the progression of heart remodeling after MI. Given our evolving understanding of Sch B and cardiovascular diseases, Sch B therapy could be effective in ischemic heart diseases as a window of opportunity.
